# Preclinical evaluation of EPHX2 inhibition as a novel treatment for inflammatory bowel disease

**DOI:** 10.1371/journal.pone.0215033

**Published:** 2019-04-19

**Authors:** William C. Reisdorf, Qing Xie, Xin Zeng, Wensheng Xie, Neetu Rajpal, Bao Hoang, Mark E. Burgert, Vinod Kumar, Mark R. Hurle, Deepak K. Rajpal, Sarah O’Donnell, Thomas T. MacDonald, Anna Vossenkämper, Lin Wang, Mike Reilly, Bart J. Votta, Yolanda Sanchez, Pankaj Agarwal

**Affiliations:** 1 Computational Biology, GlaxoSmithKline, Collegeville, Pennsylvania, United States of America; 2 Target & Pathway Validation, GlaxoSmithKline, Collegeville, Pennsylvania, United States of America; 3 Exploratory Biomarkers, GlaxoSmithKline, Collegeville, Pennsylvania, United States of America; 4 Research Statistics, GlaxoSmithKline, Collegeville, Pennsylvania, United States of America; 5 Centre for Digestive Diseases, Royal London Hospital, Barts Health NHS Trust, London, United Kingdom; 6 Centre for Immunobiology, Blizard Institute, QMUL, London, United Kingdom; 7 Pattern Recognition Receptor DPU, GlaxoSmithKline, Collegeville, Pennsylvania, United States of America; 8 Stress and Repair DPU, Respiratory Therapy Area, GlaxoSmithKline, Collegeville, Pennsylvania, United States of America; University of Roehampton - Whitelands College, UNITED KINGDOM

## Abstract

Epoxyeicosatrienoic acids (EETs) are signaling lipids produced by cytochrome P450 epoxygenation of arachidonic acid, which are metabolized by EPHX2 (epoxide hydrolase 2, alias soluble epoxide hydrolase or sEH). EETs have pleiotropic effects, including anti-inflammatory activity. Using a Connectivity Map (CMAP) approach, we identified an inverse-correlation between an exemplar EPHX2 inhibitor (EPHX2i) compound response and an inflammatory bowel disease patient-derived signature. To validate the gene-disease link, we tested a pre-clinical tool EPHX2i (GSK1910364) in a mouse disease model, where it showed improved outcomes comparable to or better than the positive control Cyclosporin A. Up-regulation of cytoprotective genes and down-regulation of proinflammatory cytokine production were observed in colon samples obtained from EPHX2i-treated mice. Follow-up immunohistochemistry analysis verified the presence of EPHX2 protein in infiltrated immune cells from Crohn’s patient tissue biopsies. We further demonstrated that GSK2256294, a clinical EPHX2i, reduced the production of IL2, IL12p70, IL10 and TNFα in both ulcerative colitis and Crohn's disease patient-derived explant cultures. Interestingly, GSK2256294 reduced IL4 and IFNγ in ulcerative colitis, and IL1β in Crohn's disease specifically, suggesting potential differential effects of GSK2256294 in these two diseases. Taken together, these findings suggest a novel therapeutic use of EPHX2 inhibition for IBD.

## Introduction

Ulcerative colitis (UC) and Crohn’s disease (CD)–the major types of inflammatory bowel disease (IBD)—are immunologically mediated chronic diseases that affect some 1–2 million people each in the US and Europe [1]. Both UC and CD are progressive diseases, with periods of remission and relapse. While the inflammation in UC is confined to the large intestine, and affects the mucosa in a continuous fashion, in CD inflammation is usually confined to the ileum and cecum and is typically transmural. Clinical symptoms presented by both diseases include diarrhea, fever, fatigue, abdominal cramping, bloody stools, reduced appetite and weight loss. Current standard of care often involves several rounds of treatment (further discussed below) as well as hospitalization, with eventual surgery, when existing medications become ineffective and/or not tolerated. This creates significant social and productivity burdens which adversely impact the patient’s quality of life.

Mesalazine (5-aminosalicylic acid, 5-ASA) is commonly prescribed for patients with mild to moderately active UC [2,3], but has shown mixed efficacy results in CD patients. European guidelines discourage its use in CD [4]. Corticosteroids are useful for short-term treatment of active CD and UC, but corticosteroid-dependent disease frequently arises, and adverse systemic effects often limit tolerability [5]. Thiopurines are useful in steroid-sparing or steroid-free maintenance of remission in both UC [6] and CD, to a certain extent [7]. Methotrexate has also been used as a steroid-sparing agent in CD but has shown little effect on UC [5]. For steroid-refractory UC, cyclosporine or anti-TNF agents (e.g. infliximab) can be given as rescue therapy [8]. Despite initial responses in 50–60% of IBD patients receiving anti-TNF, only about one third maintain clinical remission beyond the first year [9]. For many patients, surgery eventually becomes necessary. Clearly, there is a need for novel therapies beyond the currently approved small molecule and biologic treatments.

**Fig 1:**
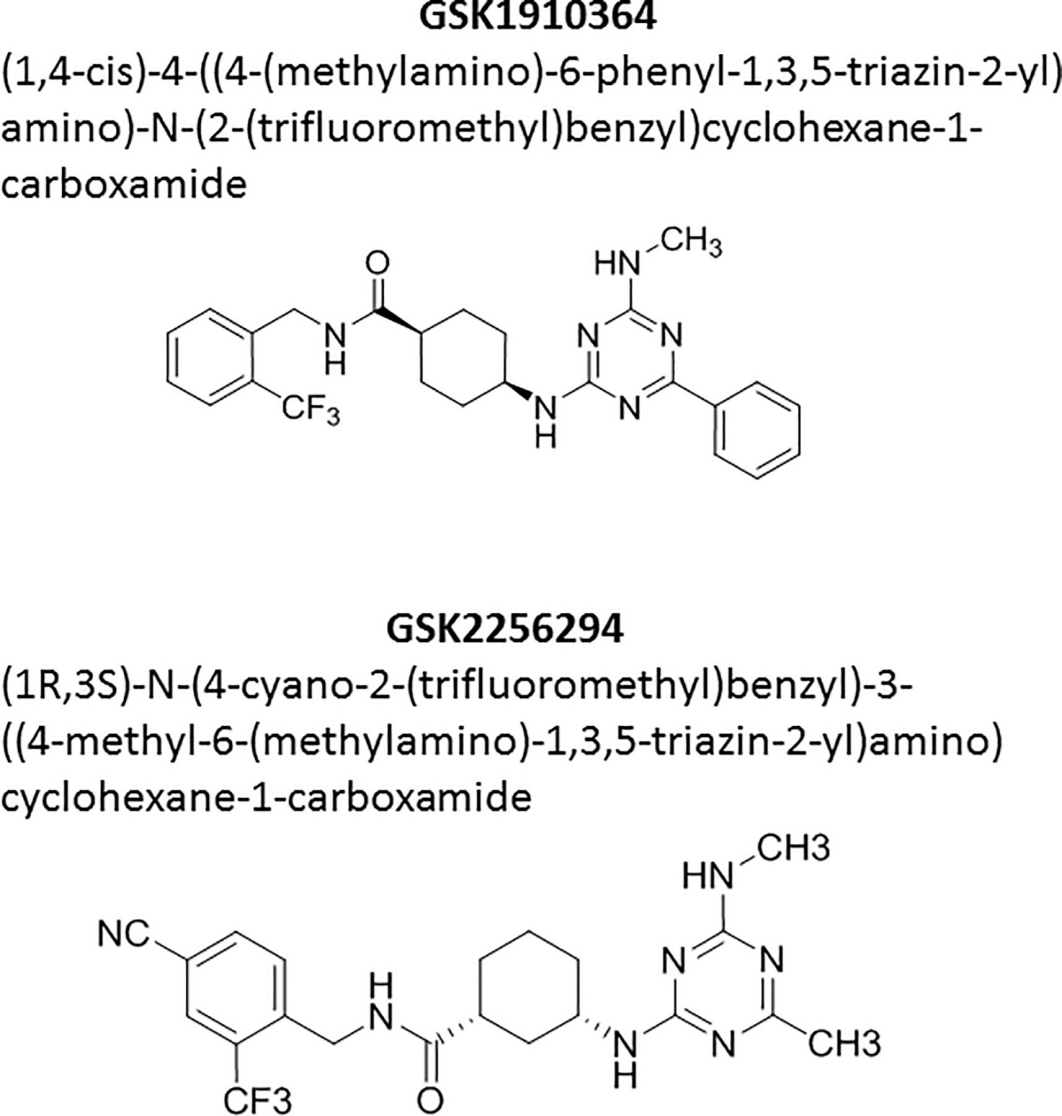
Name and structure for GSK1910364 and GSK2256294. IUPAC names and molecular structures (ChemDraw) are given for the two sEHi compounds used in the experiments described in this publication.

As part of our research efforts aimed to finding novel disease therapies, we have developed computational approaches including CMAP-based data generation as well as *in silico* data mining for drug targets with a collection of GSK clinical assets. Briefly, CMAP [10] is a computational method to compare gene expression profiles derived from compound treatment of cell lines, with gene expression signatures of diseased tissue obtained from patients or animal models. Compound profiles with strong inverse correlation to disease signatures are interesting, suggesting that compound treatment may potentially be useful for treating the disease [11]. Our data pointed us to a cluster of significant inverse correlations linking GSK2256294 (Fig 1), a soluble epoxide hydrolase inhibitor (EPHX2i), to patient-derived IBD signatures (more detail in the methods section). EPHX2 tool inhibitors have been reported to inhibit colitis and ulcer formation in dextran sodium sulfate (DSS)-induced, and in IL10 (interleukin 10) knockout mouse IBD models [12,13]. Further supporting our hypothesis, IL10/EPHX2 double knockout mice showed lower ulcer incidence and transmural inflammation, along with significantly decreased neutrophil infiltration and decreased levels of Tnf (tumor necrosis factor alpha) and Ifng (interferon gamma), compared to IL10 knockout mice [13]. IL10/EPHX2 double knockout mice also showed reduced IBD-associated tumor development [14]. In human patients, EPHX2 protein expression was elevated in tissue samples from UC, as well as from UC-associated dysplasia and colitis-induced carcinoma [15].

More recently, a GWAS study across several populations identified 38 novel IBD loci, including SNP rs17057051 on chromosome 8, in a locus containing the genes *PTK2B* (protein tyrosine kinase 2 beta), *TRIM35* (tripartite motif containing 35) and *EPHX2* [European odds ratio 0.94, European P-value 5.5 E -08] [16]. This potential human genetic association is currently of borderline genome-wide significance, but the direction is protective and consistent with known biology of EPHX2. GWAS support linking EPHX2 with IBD should be considered in the context that to date, the total number of SNPs and loci associated with IBD risk are 231 and 200, respectively. This suggests that multiple genetic drivers and environmental variables contribute to the development and progression of IBD.

Arachidonic acid metabolism generates a variety of bioactive lipids, including the well-known prostaglandins and leukotrienes. Another metabolic branch in man produces EETs via the action of cytochrome P450 enzymes CYP2C8 and CYP2J2. EETs have numerous pleiotropic effects, which include anti-inflammatory, vasoactive and analgesic properties [17]. EPHX2 hydrolyzes lipid epoxides to their corresponding diols, dihydroxyeicosatrienoic acids (DHETs), which are less lipophilic, less biologically active and more easily removed from the site of action and excreted [18]. Inhibiting EPHX2 delays the breakdown of EETs, prolonging their beneficial effects. We have developed EPHX2 inhibitors for cardiovascular and respiratory indications. The most clinically advanced molecule, GSK2256294, was discovered using DNA-encoded library technology [19]. Although studies of cardiac remodeling in rodents showed insufficient efficacy [20], inhibition of the enzyme showed protective effects in a cigarette smoke exposure mouse model of COPD [21]. In phase I trials in healthy male subjects and overweight smokers, GSK2256294 was well-tolerated and showed no serious adverse events (SAEs). The most frequent AEs were headache and contact dermatitis [22,23]. Our own research as well as emerging evidence suggest that EPHX2 inhibition might contribute to the therapeutic resolution of IBD. Hence, we decided to investigate the effects of EPHX2 inhibition in an IBD model system and in human derived disease tissue.

## Materials and methods

### CMAP

Transcriptional response profiles of GSK small molecule compounds were generated on the following platforms:

1. Illumina: In total, 636 bioactive small molecules from GSK compound collection were tested at 10 μM concentration primarily from MCF7 cell line. Each instance has attributes such as perturbagen name, concentration, cell line and batch. Probe level data was processed using Array Studio (Omicsoft Corporation, Research Triangle Park, NC, USA). Briefly, microarray datasets were grouped based on the cell line. For each microarray dataset, the probe set intensities were normalized using Quantile normalization procedure followed by batch correction to minimize any technical variation between samples. Finally, the log2 intensities of each probe set from all DMSO control samples within the same batch and cell line are averaged and subtracted from the treatment sample to generate the corresponding treatment-to-control values.
2. L1000: Over 400 GSK compounds were evaluated on the L1000 high-throughput gene expression profiling platform [24], some at multiple doses, in up to 11 cell lines. L1000 measures the expression of 978 landmark transcripts and uses a computational model to infer the expression of the remaining approximately 19,000 transcripts. The model was trained on historical gene expression profiles from published literature [25,26]. Crude cell lysates in a 384-well plate format were processed at the Genometry facility (http://www.genometry.com) using their computational pipeline. Intensity measurements for each sample were evaluated first to assess relative expression consistency of control genes. All samples that passed well- and plate-level quality thresholds were scaled, normalized and log transformed. Finally, a robust z-scoring method was used to compute differential expression values from disease profiles.

Disease expression signatures were generated from NextBio (since acquired by Illumina in 2013) and GeneLogic (Gaithersburg, MD–filed for Chapter 11 in 2011) [27]. We required that tissues have at least 3 normal and 3 disease samples for use in signature generation. Each disease signature consists of the 250 most up-regulated and 250 most down-regulated genes, selected by fold change between disease and normal tissues. Particularly interesting was a patient-derived laser-capture microdissection signature from colon epithelial cells of (N = 6) UC patients, compared to (N = 11) normal controls from GeneLogic. The signature genes are listed in S1 Table.

### DSS-induced colitis model

GSK contracted with Syngene International, Ltd. (Bangalore, India) to manage the mouse study. Every effort was made to minimize or eliminate pain and suffering of all the animals in the study. Animal care complied with the recommendations of Committee for the Purpose of Control and Supervision of Experiments on Animals (CPCSEA), Government of India and Association for Assessment and Accreditation of Laboratory Animal Care International (AAALAC). The study was also approved by the Office of Animal Welfare, Ethics and Strategy at GSK (Submission number S001744, Reference number D00010061). Female C57BL/6 mice (8–10 weeks; 18-23g) were maintained in a controlled environment with 22 ± 3°C temperature, 50 ± 20% humidity, a light/dark cycle of 12 hours each and 15–20 fresh air changes per hour. Animals were housed group-wise (3 to 5 animals per cage) and autoclaved corncob was used as a bedding material. The animals were fed, *ad libitum*, with certified Irradiated Laboratory Rodent Diet (Nutrilab brand, Tetragon Chemie Pvt. Ltd., Bangalore). Potable water, filtered through reverse osmosis, was autoclaved and provided *ad libitum* to all animals via polycarbonate bottles fitted with stainless steel sipper tubes. All the mice were kept under acclimatization for a period of about 5–7 days before initiation of the experiment. One day prior to treatment, mice were randomized based on body weight. The weight variation of the mice did not exceed 20% of the mean body weight in a group at the time of randomization. Also, it was ensured that there were no significant changes in the body weights between groups by performing statistical analyses using one-way ANOVA, followed by Dunnett’s test. On receipt, the animals were assigned a temporary number at the base of tail using marker pen. Immediately after randomization, the animals were assigned a permanent number by ear notching. Cages were identified by cage cards indicating the study number, study code, group number, sex, dose, cage number, number of animals and animal number details. Experiment design included a power analysis to determine treatment group sizes. The original study contained 15 groups (sham, DSS, CsA and 13 compound treatments representing different mechanisms). In this report we describe analysis of a subset of the original experiment. Four treatment groups were defined: Sham control (N = 6) represented the disease-free baseline, DSS-alone (N = 12) represented the untreated disease baseline, Cyclosporine A (CsA) (N = 6) represented a positive control treatment known to ameliorate disease, and sEHi (N = 6) represented the test substance of interest. The sham control group received regular drinking water throughout the study. Colitis was induced in all other groups by providing Dextran Sodium Sulfate (DSS) in drinking water at 2.5% w/v concentration from day 0 to 5. After day 5, DSS water was replaced with normal drinking water up to day 9. Compound treatments lasted from day 0 to day 9. Cyclosporine was purchased from Panacea Biotech Ltd., and 10 mg/kg was administered via i.p. injection with saline as a vehicle. sEHi GSK1910364A (Fig 1) was prepared as a suspension in 0.5% methylcellulose and dosed at 50 mg/kg orally twice daily. Body weights were recorded individually for all animals at receipt, day of randomization, prior to treatment, and daily thereafter. The animals were monitored daily, with stool consistency as well as gross bleeding recorded. A score increasing from a value of 0 to 4 was assigned to assess the extent of inflammation in the colon [28].

To measure the extent of inflammation, the Disease Activity Index (DAI), defined in Table 1, was calculated based on the formula, *DAI = (Body weight score + Stool consistency score + Fecal blood score)/3*

**Table 1:** DAI scoring system for DSS model.

Score	Weight Loss (% day0 weight)	Stool Consistency	Blood in Feces
0	<1% reduction in body weight	Normal pellets	No blood
1	≥1% <5% reduction in body weight		
2	≥5% <10% reduction in body weight	Loose feces	Visible blood in feces
3	≥10% <15% reduction in body weight		
4	>15% reduction in body weight	Diarrhea	Bleeding from the rectum

All animals completed the study, and on day 9 the mice were euthanized using CO_2_ asphyxiation under isoflurane anesthesia, and colon tissues were isolated from ileo-cecal junction to rectal end. The colon was cleaned with PBS to remove the fecal matter. Length and weight of each colon were recorded. One third of colon (proximal) was preserved in 10% neutral buffered formalin (NBF) and processed for histopathology. Second one third (middle portion) was weighed and flash frozen in liquid nitrogen and preserved in -80°C for myeloperoxidase (MPO) assay. Remaining one third of colon (distal end) was weighed and flash frozen in liquid nitrogen and stored at -80°C.

For colon homogenization, 50mM potassium phosphate buffer, pH 6.0 containing 0.5% HTA-Br and 10mM EDTA was used. The colon tissues were homogenized in 500μL of buffer and the final volume made up to 1 ml. The homogenates were subjected to two rounds of freeze-thaw followed by a brief sonication of 10s. The samples were then centrifuged at 13,000 rpm for 10 minutes and the clear supernatants were assayed for MPO activity using a traditional colorimetric assay using dianisidine dihydrochloride substrate. The MPO activity was normalized to total protein levels in the homogenate and expressed as MPO per g of colon tissue.

### Mouse colon histopathology

For histopathological analysis, sections of colon were prepared and processed by routine histopathological processing. Paraffin blocks were made and 4–5μm thick tissue sections were prepared on glass slides using an automated rotary microtome. The slides were stained with hematoxylin and eosin (H&E) and examined under the microscope for semi-quantitative scoring. The stained colon tissues were analyzed for microscopic changes related to DSS administration under Nikon Eclipse 80i microscope. Representative photographs were taken using Leica DFC425C camera attached to the microscope. Table 2 depicts the criteria used for histopathology scoring, based on [29].

**Table 2:** Histopathology scoring system for DSS model. Histopathology Scoring Pattern (Scale 0–40).

Feature	Severity Score & Description	Area Score Based on Percentage Affected
Inflammation	0 None	
	1 Slight	
	2 Moderate	
	3 Severe	Score 1: 1–25% area involved
Extent of Damage	0 None	Score 2: 26–50% area involved
	1 Mucosa	Score 3: 51–75% area involved
	2 Mucosa and submucosa	Score 4: 76–100% area involved
	3 Transmural	
Crypt Damage	0 None	
	1 Basal 1/3 Damaged	
	2 Basal 2/3 Damaged	
	3 Only surface epithelium intact	
	4 Entire crypt and epithelium lost	

### RNA extraction and RT-PCR of mouse colon tissue

Mouse colon tissue were kept frozen and stored at -80 C. RNA extraction was performed using QIAzol Lysis Reagent (200 ml) (cat. no. 79306), MaXtract High Density (200 x 2 ml) (cat. no. 129056) according to the manufacturer’s instructions. We found that the DSS treated samples failed to produce results in the follow up RT-PCR steps. RNA sample were further purified through the Turbocapture RNA purification kit (Cat# 72271, Qiagen) for additional cleanups. Purified mRNA was quantified by NanoDrop spectrophotometer (ThermoFisher) and stored in -80 C. 12μg of RNA were reverse transcribed into cDNA using the High Capacity cDNA Reverse Transcription kit (Cat. No. 4368814, ThermoFisher) in a 40μl total reaction volume on 96-well plate. Reverse transcription was performed at 37 C for 2hrs in a Thermocycler. PCR reactions were setup using TaqMan Universal Master Mix II (Cat. No. 4440043, ThermoFisher) in on a 384-well plate in 5μl reactions/well. cDNA (500nl/reaction) and TaqMan primers (250nl/reaction) were stamped into the reaction plate containing the master mix using the Echo 555 acoustic dispenser (Labcyte). The Real Time quantitative PCR were performed in the Lightcycler 480 (Roche).

### RT-qPCR data analysis

The Cps (Crossing Point) obtained from the Lightcycler 480 were converted to ΔCp by normalizing to house-keeping gene GAPDH using the formula ΔCp = Cp GAPDH—Cp Sample. ΔCp value was further normalized to the sham treated control to obtain ΔΔCp using the formula ΔΔCp = ΔCp DSS or compound treated - ΔCp sham treated. The data represent the average of 4 biological repeats of the mouse tissue and 2 technical duplicates of each RT-PCR reactions.

RT-PCR primer list: Tnf mm00723601; Il1b mm00434228; Ccr6 mm99999114; Sstr2 mm00436685; Cdh3 mm01249209; Ltb mm00434774; Rela mm00501346; Lgr5 mm00438890; Gapdh mm99999915.

### IHC of normal and diseased human colon tissue samples

Materials:

Formalin fixed paraffin embedded tissue samples were sourced ethically, and their research use was in accordance with the terms of the written informed consent document (Protocol No. MTG-022, IRB ID 3764–001 Sterling IRB, June 2011).

10 Crohn’s biopsy samples

1 Ulcerative colitis.

1 normal colon

1 skeletal muscle (negative control).

HEK539 sEH BacMam transduced and non-transduced cell pellets used as positive controls.

Reagents: Discovery Anti Ms HQ, cat 760–4815, Ventana Medical Systems

Discovery Anti HQ–HRP, cat 760–4820, Ventana Medical Systems

Discovery Purple, cat 760–229, Ventana Medical Systems

Discovery Inhibitor, cat 760–4840, Ventana Medical Systems

Hematoxylin II, cat 790–2208, Ventana Medical Systems

Bluing Reagent, cat 760–2037, Ventana Medical Systems

Method: Mouse monoclonal antibody clone 1A6 was used to stain for sEH in human normal and diseased gut tissue sections. Clone 1A6 was selected for displaying specificity using BacMam sEH transduced HEK539 cells in addition to positive (ulcerative colitis biopsy) and skeletal muscle as negative control (EPHX2 tissue expression). Immunohistochemistry (IHC) was carried out on the Ventana Discovery Ultra automated staining platform (Ventana, Tucson, AZ). In brief, FFPE sections were mounted onto positively charged glass slides, air dried and baked at 60°C in an oven prior to IHC. Tissue slide sections were loaded onto the Ventana Discovery Ultra instrument (Ventana, Tucson, AZ). Deparaffinization followed by heat induced epitope retrieval was performed using Tris-based (EDTA) buffer solution, CC1 (Ventana, Tucson, AZ). Mouse mAb, 1A6 (1μg/ml, cat NBP2-02948, Novus Biologicals) or mouse Ig isotype control (1μg/ml, Invitrogen) were incubated for 40 mins at 37°C. Antibody detection was achieved with 16 minutes’ incubation with Discovery Anti mouse HQ (Ventana, Tucson, AZ), followed by another 16 minutes’ incubation with anti HQ—HRP (Ventana, Tucson, AZ). Visualization was achieved by application of Discovery Purple chromogen (Ventana, Tucson, AZ). Tissue sections were counterstained with haematoxylin. Images were captured using the NanoZoomer slide scanner (Hamamatsu, Bridgewater, NJ).

### Immunohistochemical (IHC) scoring

IHC staining was assessed using a subjective grading system (0–4: 0 = negative, 1 = minimal, 2 = mild, 3 = moderate, 4 = marked) to grade the staining intensity/distribution for specific intestine structures [mucosa, lamina propria leukocytes, endothelium, muscularis, and inflammatory infiltrates, germinal centers associated with mucosal associated lymphoid tissue (MALT) and/or inflammatory follicles, and myenteric plexi].

### Human colon biopsy explant assay

Tissue was obtained during routine endoscopy of patients with IBD. All patients took part in this study after providing informed written consent and were recruited between 12 January and 29 June, 2016. The study was approved by the local ethics review panel (NRES, East London REC 2; REC study number is 10/H0704/74). The human biological samples were sourced ethically, and their research use was in accord with the terms of the informed consent. Explant cultures were performed as previously described [30]. Colon biopsies from UC or CD patients during endoscopic examination were taken from inflamed mucosa and stored on ice. For 6 samples from each donor, two samples were cultured and treated with 0.1% DMSO or 1 μM prednisolone as controls, the other 4 samples were cultured and treated with compounds. The supernatants as well as cultured biopsies were collected for the cytokine and metabolite analysis after compound treatment for 24 hours. The release of cytokines was determined using a commercially available ELISA kits (TNFα and IL1B, BioTechne; and IL6, ImmunoTools). Statistical analysis was performed using a repeated measure mixed model approach.

UC and CD patient sample supernatants (N = 10–12) were analyzed at GSK for IFNG, IL1B, IL2, IL4, IL6, IL8 IL10, IL12p70, IL13 and TNFα protein concentrations, using the MSD method. The cytokine panel kit was from Meso Scale Discovery Cat#. K15049D02. The frozen samples (supernatants from cultured IBD biopsies, treated with or without compound) were diluted 20-fold in PBS. The detection was then followed the kit instruction. UC and CD patient sample supernatants (N = 10–12) were analyzed for IL1B, IL6 and TNFα protein concentrations by Anna Vossenkaemper, using ELISA kits as described in [31].

### Human EPHX2 protein analysis

Western blot was used to analyze the EPHX2 protein amount in human colon biopsy samples. Human colon samples were homogenized in lysis buffer to prepare colon tissue lysates. 20 μg protein of each sample lysate was used to run in a 4–12% gradient SDS-PAGE. The blot was blocked with Li-Cor Odyssey blocking buffer for 30 min at RT, then blotted with anti-human EPHX2 antibody (from Santa Cruz Biotech., Cat# sc-25795) at 1:500 dilution in the locking buffer for overnight at 4C with rotation. After 3 times washing, the blot was incubated for 1 hr at RT with goat anti rabbit antibody (IRDye800CW, from Li-Cor, Cat#926–32211) at 1:4000 dilution in the blocking buffer. After 3 times washing, the blot was scanned with Li-Cor Odyssey scanner. Anti β-actin antibody (Santa Cruz Biotech, Cat# sc-47778) was blotted as a control. The EPHX2 band intensity was normalized to actin band intensity in the final analysis.

### Statistical analysis

The planning for the primary study comparisons with the DSS induced rodent colitis model was based on the use of summary responses (DAI, which combined three colitis related responses, and AUC, which combined the responses across the study period). The analyses of the EPHX2i small molecule treatment differences from the vehicle treatment were part of a larger treatment screening study with the DSS colitis model. The total screening study included the four study groups reported in the Results section (a sham control treated group, plus three DSS induced groups with control, CsA, and EPHX2i treatments) plus 11 additional non-EPHX2i small molecule screening treatments. The total screening study was designed to reliably detect a treatment difference of the DAI AUC of about half that seen in previous studies with the CsA positive control (80% power to detect a difference with a single-sided p-value of 0.05). The comparison of the AUC responses from the DSS control and the EPHX2i treatment groups used the estimate of the difference of the LSMeans for the treatments in an ANOVA model with the 14 DSS induced treatment groups in the total screening study.

The study with the human colon biopsy samples used multiple tissue punch samples from the donor colon. With this type of repeated sampling from the same donor, the analysis required a repeated-measures mixed model analysis by protein. The mixed model approach also provided a tool for properly weighting the individual sample vehicle and EPHX2i treatment responses within a donor and between donors. Each of the assay methods had a separate set of analyses (i.e. separate analysis models for the same protein expression comparison). Previous work with inflammation related protein expression comparisons led to the use of log transformed values for these analyses.

A review of the sample material acquisition revealed the potential for a confounding of the accrual time periods with the treatment levels assigned to the specific tissue samples. An assessment of the potential importance this confounding used a random effect model based on four sample accrual time periods. The model selection (to include or to remove the accrual period random effect) applied the Akaike information criterion (AIC) methods.

The analysis of the treatment differences for the DSS induced colitis model and the human colon biopsy sample assays both used the SAS version 9.3 Mixed Procedure.

## Results

### Connectivity MAP analysis highlights soluble epoxide inhibition as a mechanism of interest to reverse IBD disease signatures

Connectivity map (CMAP) [10] systematically compares gene expression profiles from compound treatment of cell lines, with gene expression signatures of diseased tissue from patients or animal disease models. The premise is that strong inverse-correlations at the transcriptomics level between compound and disease profiles imply that the mechanism represented by that compound may act to restore disease expression toward a more normal pattern. Thus, specific mechanisms identified from such computational analyses may be further investigated for disease relevance. We created transcriptomic profiles for a library of several hundred GSK compounds across a number of human cell lines and primary cells, as part of a computational approach for systematic target and asset prioritization as well as for potential drug repurposing programs. These profiles were compared to disease signatures assembled from both public-domain and proprietary sources (NextBio, GeneLogic). As part of our *in-silico* data mining, we observed a cluster of significant hits (inverse correlations) for EPHX2i GSK2256294, when compared to patient-derived IBD signatures. Hits to UC signatures were more frequent than to CD signatures and were more frequently related to tissue biopsy samples than to samples from blood cells such as PBMCs. We also observed highly significant hits to a laser capture microdissection (LCM)-derived signature of colon epithelial cells from UC patients (N = 6) compared to normal subjects (N = 11), available from GeneLogic. The LCM technique allows for very precise detection and collection of diseased and normal epithelial cells, with minimal contamination from other infiltrating immune cell types, and the EPHX2i compound-to-LCM signature hits were among the best hits of any compound in the collection to those disease signatures. The processed disease signature, containing the top 250 transcripts over- and under-expressed compared to normal cells, is listed in S1 Table. Consistent with the observations described above, our data analysis also identified significant hits for GSK2256294 to public domain rodent signatures derived from the DSS-colitis model (S2 Table).

### Treatment with EPHX2 inhibitor GSK1910364A reduced the disease activity index score in DSS-induced colitis

**Fig 2:**
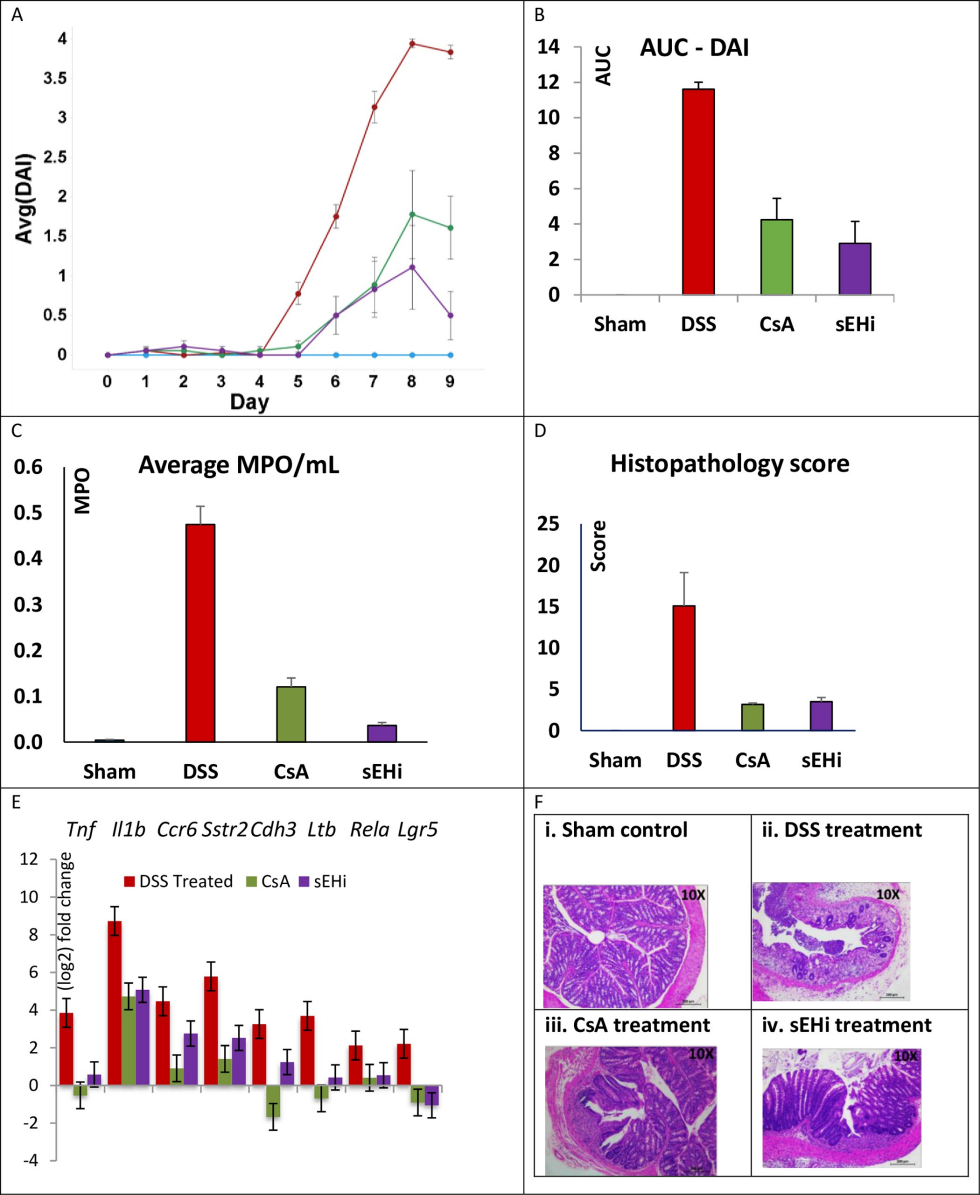
DSS-induced colitis mouse model. DSS-induced colitis is a common in vivo model for IBD. Our study employed acute dosing of DSS from Day 0 to Day 5. Compound treatments were from Day 0 to Day 9. Experimental groups were: Sham = vehicle control (no DSS or other treatment, n = 6), DSS = DSS-only (n = 12), CsA = 10 mg/kg i.p. treatment (n = 6), EPHX2i = GSK1910364A 50 mg/kg orally, twice daily (n = 6). (**A**) Mean DAI is plotted versus time; error bars reflect standard errors. (**B**) AUC values for each group were calculated at experiment conclusion. Sample sizes for each group are the same as in (A). Both CsA and EPHX2i significantly decreased the AUC compared to DSS-only control (ANOVA P-value < 0.05). (**C**) MPO protein expression is used as a surrogate biomarker for inflammation. MPO was nearly absent in the sham control mice, but levels were significantly increased in the DSS-treated animals. Both cyclosporine and EPHX2i treatment significantly decrease MPO expression, with EPHX2i showing a return to near normal level. (**D**) The histopathology scoring system is given in Table 2. DSS-treatment significantly increased the score, compared to vehicle control group. Both CsA and EPHX2i treatment significantly decrease the histopathology score by similar amount. (**E**) RT-PCR measurement of selected marker gene mRNA expression. All expression values were normalized to the levels in the vehicle control group. For most marker genes, DSS-treatment caused large increases/decreases in mRNA levels. Both CsA and EPHX2i treatment tended to restore expression levels back toward baseline (*Il1b*, *Ccr6*, *Sstr2*, *Lgr5*) or to near baseline levels (*Tnf*, *Ltb*, *Rela*). (**F**) H&E staining of selected colonic tissue sections from DSS-mice. 10x magnification of representative colon sections from each of the four treatments groups. (**i**) the sham control shows normal histology. (**ii**) the DSS-control shows widespread damage. (**iii**) CsA and (**iv**) EPHX2i treatment both show substantial protection from DSS-induced damage.

To further validate our *in-silico* findings from CMAP, we turned to an *in vivo* rodent model–DSS-induced colitis. In the acute form of the model, DSS is dosed for five days and then removed [32]. Treatment induces ulcers, submucosal edema and other histopathological lesions, along with weight loss and bloody diarrhea–resembling human UC [33]. The Disease Activity Index (DAI) score measures the combined severity of body weight loss, diarrhea and rectal bleeding induced by DSS treatment, and was assessed as summarized in Table 1. DAI time-course results for our study are shown in Fig 2A. In the sham control group (n = 6), DAI remained at zero throughout the study, while the mean score for the DSS group (n = 12) began to rise on day 5 and peaked on day 8, before declining on day 9. Cyclosporin A (CsA), with efficacy demonstrated previously in the DSS-colitis model, was chosen as a positive control [34–36]. In our study, CsA treatment (10 mg/kg i.p., n = 6) showed protection, with mean DAI peaking at 1.8 on day 8. Treatment with the pre-clinical EPHX2i GSK1910364A (50 mg/kg oral, twice daily, n = 6) offered even greater protection, with a mean DAI peak of 1.1 on day 8.

An alternate way to visualize these type of results is to compare the area under the curve (AUC) for each treatment group, as shown in Fig 2B. Low AUC values represent a protective effect. In our experiment, both CsA and EPHX2i reduced DAI AUC significantly, compared to DSS alone.

To better illustrate individual animal variation, we present per animal data points for DAI time course and AUC in S1A Fig. and S1B Fig. DSS treatment resulted in reduced colon length, increased colon weight and a corresponding increase in weight to length ratio, as compared to sham control (S2 Fig.). CsA treatment partially reversed all these trends. Interestingly, EPHX2i treatment partially reversed the colon shortening, but we observed a large increase in colon weight, leading to an increased weight to length ratio.

Myeloperoxidase (MPO) activity is a marker for inflammation, and fecal MPO levels have been proposed to reflect treatment outcome in IBD patients [37]. Therefore, MPO activity per gram of colon tissue was used to assess inflammation associated with neutrophilic infiltration into the colon. MPO activity for the untreated DSS group was 5.64, compared to 0.15 for the sham control. CsA reduced MPO activity to 1.21, and remarkably, EPHX2 inhibition by GSK1910364 further reduced MPO activity to 0.33 (Fig 2C). The proximal end of dissected colon was examined for histopathological changes, and scored as described in Table 2, using a scale of 0–40. For the DSS group, the histopathological score was 15.1 ± 4.0. Both the CsA and EPHX2i treatment groups showed evidence of protection, with scores of 3.2 ± 0.2 and 3.5 ± 0.5, respectively (Fig 2D). Individual animal values for histopath score are illustrated in S1C Fig.

### EPHX2 inhibitor GSK1910364A decreased proinflammatory cytokine production in colonic tissue of DSS treated mice

Both EPHX2i and CsA treatments resulted in down-regulated expression of proinflammatory markers *Tnf*α, *Il1b* (interleukin 1 beta), *Ccr6* (C-C motif chemokine receptor 6) and *Rela* (v-rel reticuloendotheliosis viral oncogene homolog A), suggesting both cytoprotective and anti-inflammatory effects mediated by EPHX2i treatment (Fig 2E). We also observed decreased mRNA expression of *Ltb* (lymphotoxin B), *Lgr5* (leucine rich repeat containing G protein coupled receptor 5), *Cdh3* (cadherin 3), and *Sstr2* (somatostatin receptor 2) in the CsA and EPHX2i groups, compared to DSS alone. These markers also show increased gene expression in human IBD patient tissue: *LTB*, *LGR5*, *CDH3*, and *SSTR2* [38–41].

Fig 2F shows Hematoxylin and Eosin (H&E) staining of selected tissue sections illustrating the histopathological findings, comparing similar fields (10X magnification) of representative (i.) sham control, (ii.) DSS, (iii.) CsA-treated, and (iv.) EPHX2i-treated colon groups. The sham control animals showed normal crypt structure and abundant goblet cells and villi. The DSS-treated group showed nearly complete destruction of the colonic mucosa. The crypts were extensively damaged, with a nearly complete absence of goblet cells. There were signs of severe inflammation, as evidenced by inflammatory cell infiltration of the mucosa and lamina propria. The CsA group showed less than 25% damage. Localized areas of inflammatory reaction were seen in the lamina propria. Goblet cells and glands were present but reduced in number compared to the sham control animals. The EPHX2i group also showed substantial protection. Damage was limited to a small area, with only mild inflammation evident in other areas.

### EPHX2 protein is present in normal and increased in IBD human colon tissue

**Fig 3:**
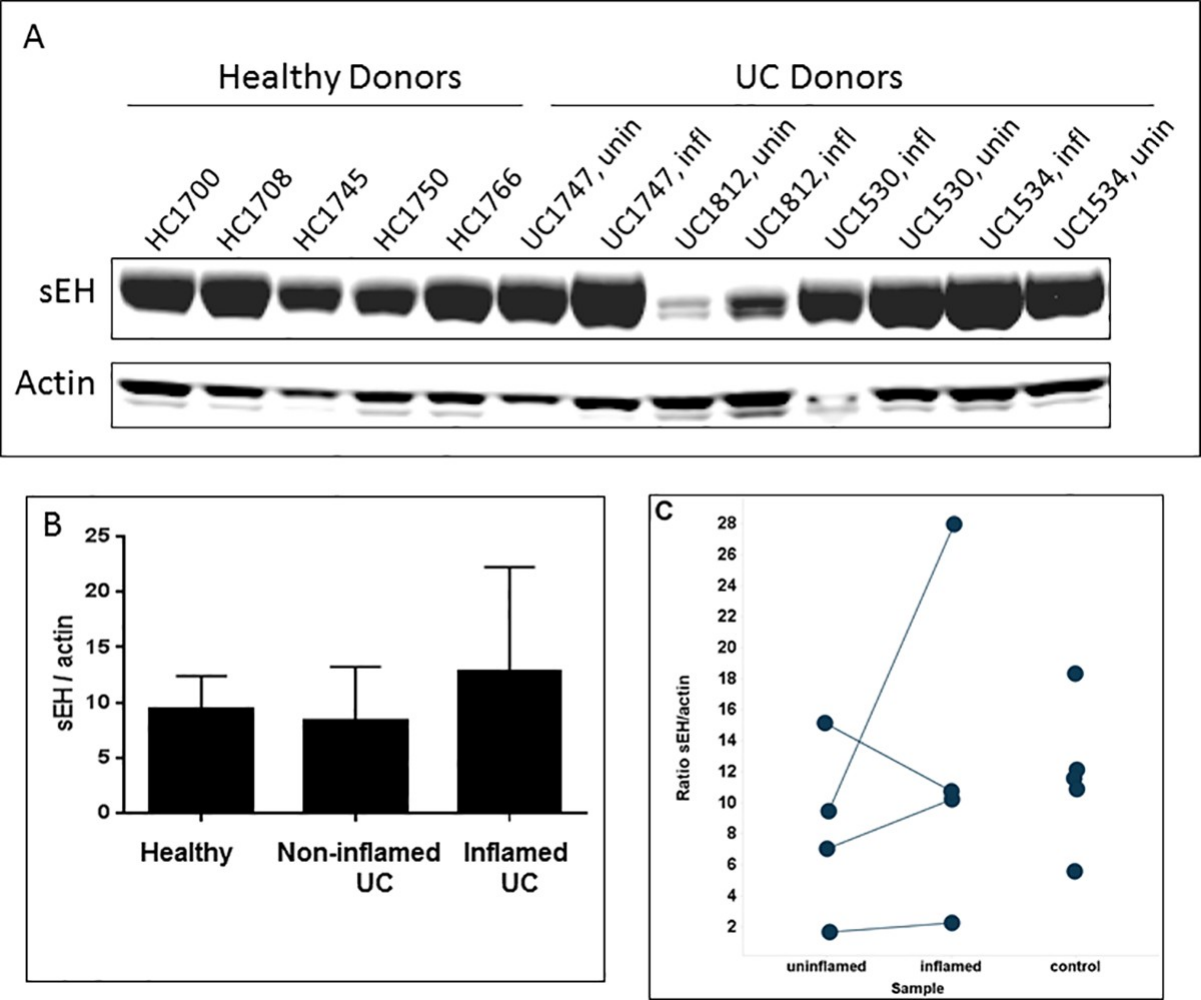
Western blot analysis for EPHX2 protein level in human colon. Human colon samples were from healthy donors or UC donors. Each UC donor had matched uninflamed (unin) and inflamed (infl) biopsies. (A) Representative western blot results. (B) Normalized mean EPHX2 intensity vs. control actin from combined donors for each group. Data derived from duplicate western blot analyses with the same set of samples. Error bars represent standard deviations. Differences between groups were not statistically significant. (C) EPHX2/actin ratio for individual donors from each sample group. Differences between groups were not statistically significant.

To bridge the gap from mouse model to human disease, and to further establish translational relevance for our hypothesis, we turned to colon biopsies from IBD patients to determine whether changes in EPHX2 protein expression are evident in the disease state. We examined EPHX2 protein expression in tissue samples from routine endoscopies of UC patients (n = 4, paired inflamed and uninflamed regions) compared to healthy controls (n = 5) using Western blot analysis, as shown in Fig 3. EPHX2 protein was detected in all samples, and in three of the four patients there was a trend toward increased expression (albeit not statistically significant), correlating with inflammation. Due to patient variability, it was difficult to assess whether increased EPHX2 protein expression trends robustly with inflammation in this dataset.

**Fig 4:**
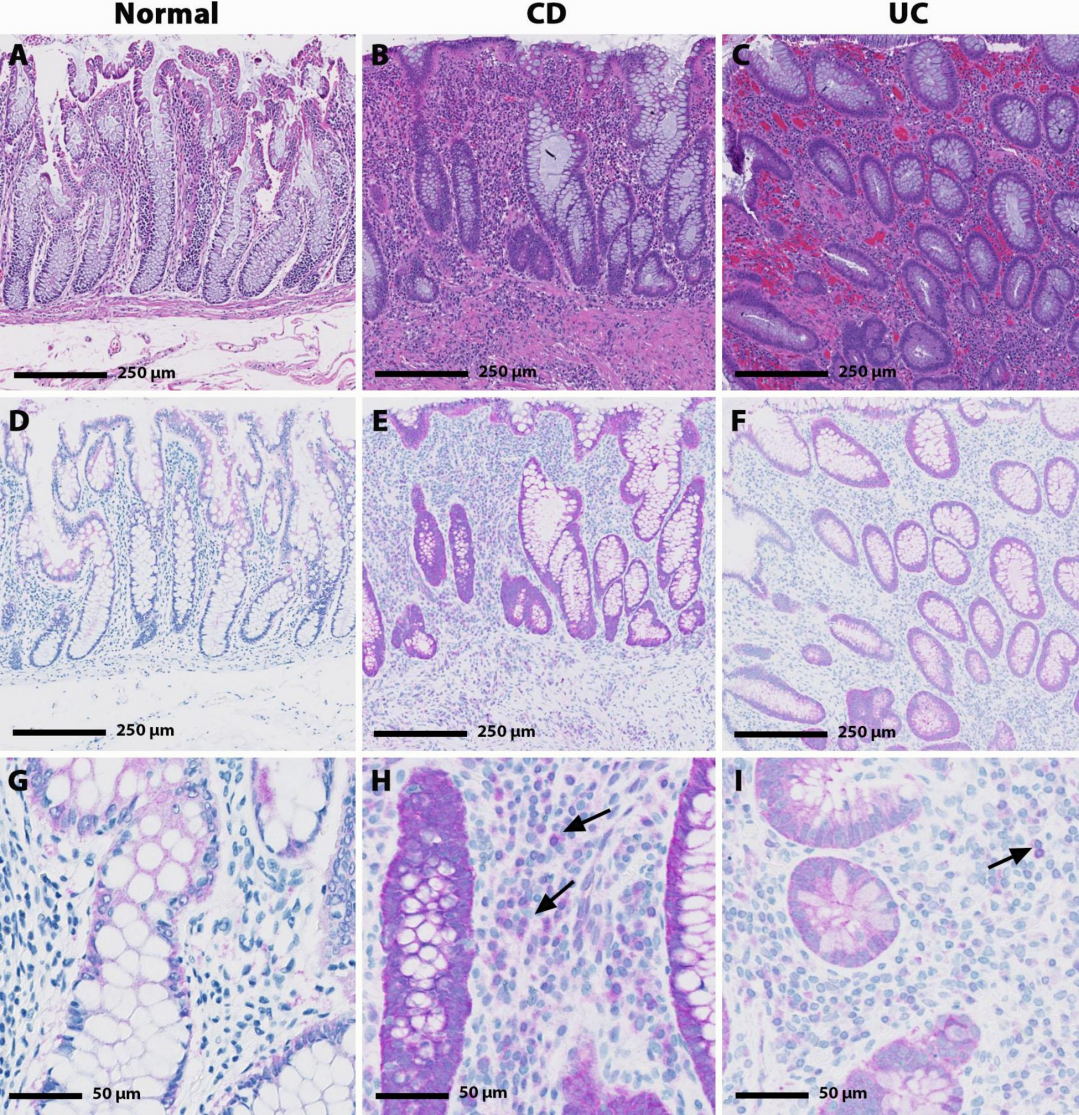
**Hematoxylin and eosin (H&E; A-C) and EPHX2 immunohistochemical (IHC; D-I) staining in human normal, CD and UC biopsy samples**. Minimal cytoplasmic EPHX2 IHC staining is present in the colonocytes of the normal colon sample (D & G) with no EPHX2 IHC staining evident in the lamina propria leukocytes. Increased EPHX2 immunoreactivity was observed in the CD and UC samples. Marked cytoplasmic colonocyte and minimal to mild lamina propria leukocyte (arrow) EPHX2 IHC staining is present in the CD sample (E & H); whereas, mild cytoplasmic colonocyte and minimal lamina propria leukocyte (arrow) EPHX2H IHC staining is present in the UC sample (F & I).

Immunohistochemistry (IHC) analysis was used to assess protein localization of EPHX2 in colon samples from healthy volunteers as well as paired uninflamed and inflamed colon samples from UC (n = 2) and CD (n = 8) patients. Representative stained samples are shown in Fig 4. The microscopical intensity of inflammation was assessed using the Global Histology Activity Score (GHAS), both in its original version [42] and in its modification (modGHAS) [43], which does not include the evaluation of the number of involved bowel segments (see Table 3). GHAS ranged from 1 to 11 for the ten patient IBD biopsies. The two UC biopsies had GHAS of 8 and 10; whereas the CD biopsies GHAS ranged between 1 and 11. Two CD biopsies (#1 and #5) did not have mucosa or lamina propria present which contributed to lower GHAS for those patients.

**Table 3:** Qualitative histopathology using the GHAS scoring system for IHC scoring.

Sample	Tissue Type	IHC score	Histopathology score (GHAS)
Normal	Colon	1	0
Crohn’s Disease	Ileum/Cecum	3	4
Crohn’s Disease	Colon	10	11
Crohn’s Disease	Ileocolic	8	1
Crohn’s Disease	Ileum/Cecum	5	4
Crohn’s Disease	Ileum/Colon	10	2
Crohn’s Disease	Colon	12	3
Crohn’s Disease	Colon	1	10
Crohn’s Disease	Colon	13	9
Ulcerative Colitis	Colon	9	8
Ulcerative Colitis	Colon	9	10

EPHX2 IHC staining in the normal human colon sample was minimal to mild in the following structures: mucosal epithelium, myenteric plexi, and mucosal associated lymphoid tissue (MALT) germinal center (Fig 4A, 4D and 4G). Compared to the normal colon, the IBD biopsies had increased EPHX2 IHC staining (Fig 4B, 4C, 4E, 4F, 4H and 4I) in the following structures: mucosal epithelium (grades 2–4 in 7 /8 patients), lamina propria leukocytes (grades 1–2 in 7 /8 patients), endothelium (grades 1–2 in 9 /10 patients), muscularis (grades 1–2 in 9 /10 patients), transmural inflammatory infiltrates (grades 1–4 in 9 /10 patients), germinal centers (grades 3–4) in 3 /4 patients), myenteric plexi (grades 2–3) in 6 /7 patients).

### Treatment with EPHX2i GSK2256294A decreased cytokine release from IBD patient-derived colon explants

**Fig 5:**
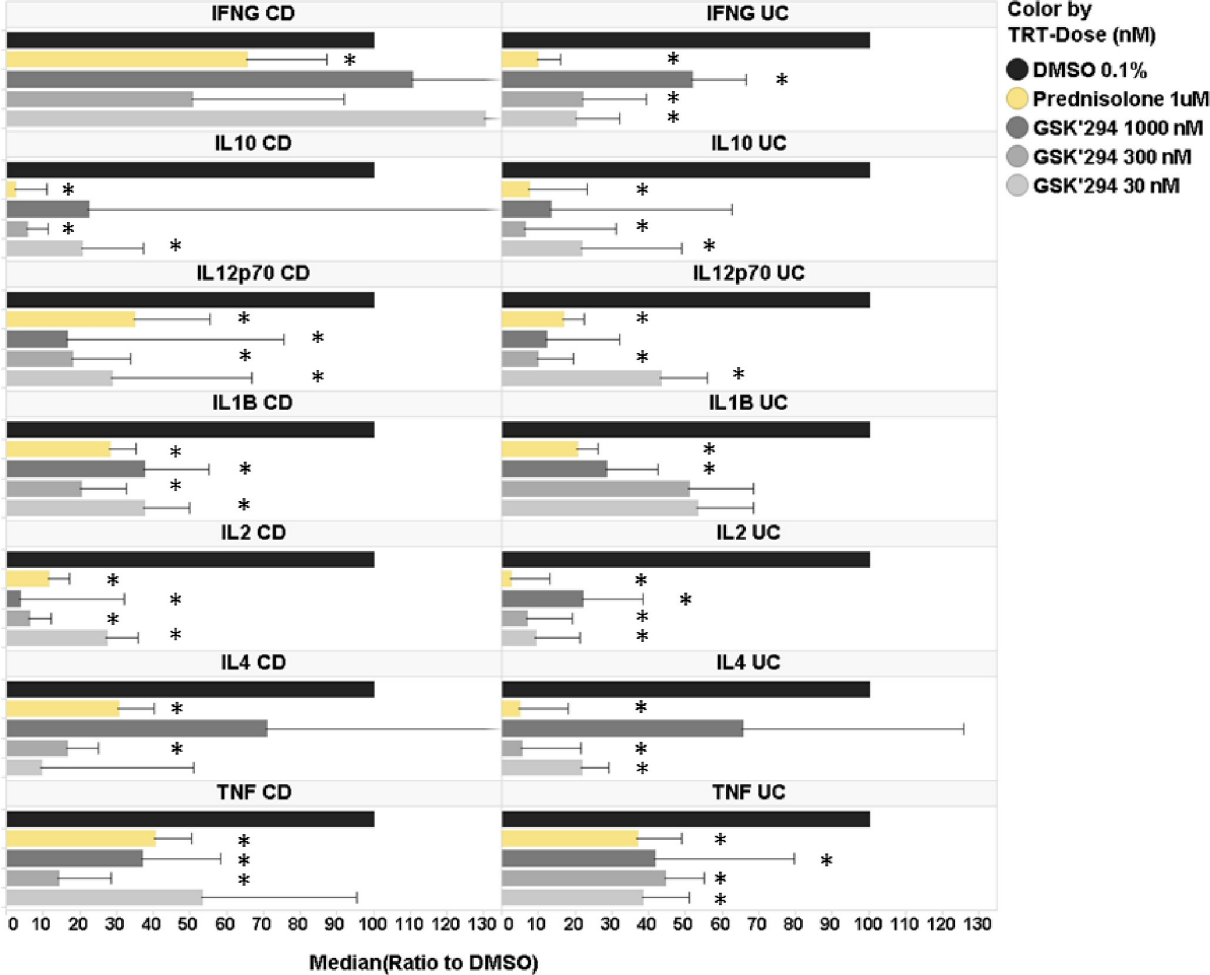
Effect of GSK2256294 on cytokine release from UC and CD colon explants measured by MSD. UC or CD tissue samples were incubated for 18 h with varying doses of GSK2256294 (GSK294), or prednisolone (positive control). GSK2256294 doses are in nM. Cytokine release in the cell-free supernatant was determined using MSD assay as described in Materials and Methods and were normalized to DMSO (negative control). Samples from N = 6 UC and N = 10 CD donors were used in the cytokine analysis. Error bars represent standard errors. * denotes p <0.05 *vs* DMSO control.

To further validate our hypothesis, we applied an *ex vivo* colon explant assay [44] for assessing the effect of GSK2256294 on spontaneous cytokine production by cultured inflamed colon biopsies freshly obtained from UC or CD patients. Cytokine release is a useful surrogate biomarker of the inflammatory processes taking place in the colonic mucosa [45]. We therefore measured the protein levels of IFNG (interferon gamma), IL1B (interleukin 1 beta), IL2, IL4, IL6, IL8, IL10, IL12p70, IL13 and TNFα in the supernatants of cultured biopsies, using MSD as described in the Methods (Fig 5). Colon explants were treated with 0.1% DMSO (negative control), 1 μM prednisolone (positive control) or varying doses of GSK2256294 (0.003, 0.03, 0.3, 3, 30, 300 and 1000 nM), although due to tissue availability not all patient samples were treated with the full dose range. Samples from ten patients with UC were analyzed, along with eight samples from CD patients. Measured values were normalized to DMSO control. As shown in Fig 5, prednisolone treatment significantly decreased protein expression of most cytokines (the exceptions were IL13 in UC samples, and IL8 in both UC and CD samples). At higher concentrations, GSK2256294 also significantly decreased protein expression of most cytokines, but the efficacy of drug treatment varied with the specific cytokine, the dose and the disease origin of the patient sample. For example, IFNg showed a significant decrease in the 0.3, 30, 300 and 1000 nM GSK2256294 groups, but only for UC samples. No significant effect was observed in the CD samples at any dose. For most of the other cytokines, there was a comparable effect of GSK2256294 in both UC and CD. However, we did notice that for several cytokines, decreased protein expression was seen with lower doses of GSK2256294 in UC than in CD samples (IL4, IL12p70 and TNFα).

**Fig 6:**
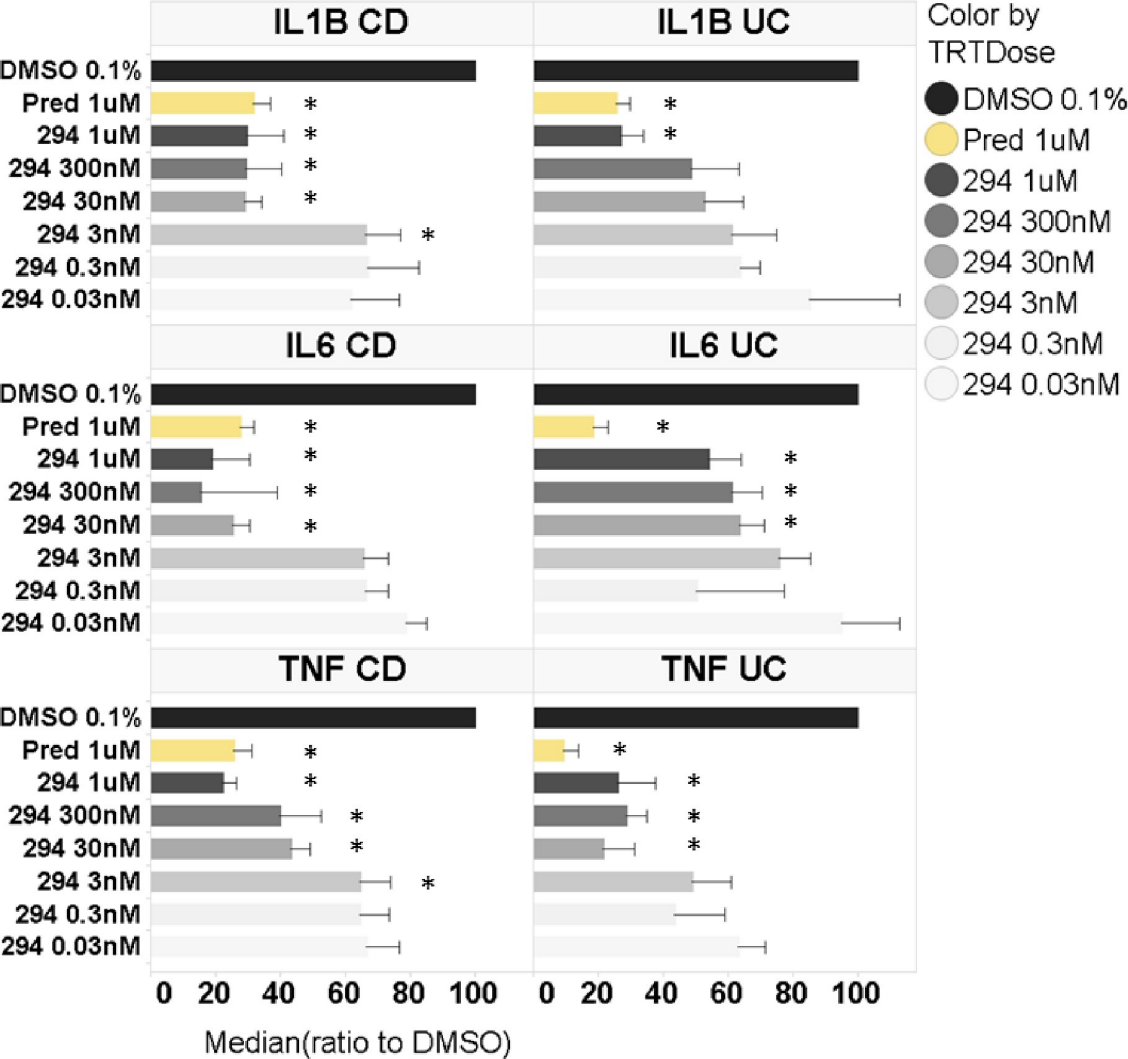
Effect of GSK2256294 on cytokine release from UC and CD colon explants measured by ELISA. UC or CD tissue samples were incubated for 18 h with varying doses of GSK2256294 (294), or prednisolone (positive control). GSK2256294 doses are in nM. Cytokine release in the cell-free supernatant was determined by ELISA as described in Material and Methods and were normalized to DMSO (negative control). Samples from N = 6 UC and N = 10 CD donors were used in the cytokine analysis. Error bars represent standard errors. * denotes p<0.05 *vs* DMSO control.

To extend these observations, we measured selected disease-relevant cytokine protein expression from the same patient samples via ELISA. Levels of TNFα, IL1B and IL6 in biopsy supernatants from UC and CD patients are shown in Fig 6. Compared to DMSO control, GSK2256294 significantly inhibited secretion of all three cytokines measured, both in UC and CD patient tissue explants. In UC patient samples, the highest dose of GSK2256294 (1 μM) inhibited IL1B release to the same extent as 1 μM prednisolone, and even 0.3 nM GSK2256294 significantly decreased IL1beta compared to DMSO. For IL6 and TNFα, 1 μM GSK2256294 appeared less effective than prednisolone, but even a concentration of 30 nM decreased cytokine levels compared to DMSO control. In the CD patient samples, doses as low as 30 nM GSK2256294 decreased IL1B and IL6 expression comparably to 1 μM prednisolone. For TNFα, only the highest dose of GSK2256294 showed similar inhibition to prednisolone.

## Discussion

Our research efforts added to emerging evidence strongly point to the potential therapeutic benefits of inhibiting EPHX2 in IBD patients. *Ex vivo* measurements in colon tissue biopsies obtained from IBD patients showed that EPHX2 inhibition can decrease secretion of proinflammatory cytokines. A recent study showed that EETs concentrations were higher in UC tissues compared to matched adjacent non-inflamed tissue [46]. UC tissues also showed reduced EPHX2 expression and increased *CYP2J2* levels, both of which would lead to increased EETs, as was observed. The relative EPHX2 expression in disease vs. normal seems to differ between our observations and those of Qiu et al [46], which might be explained by the relatively small number of samples analyzed in both studies. Differences in disease stage may also be a factor, since our samples were derived from routine endoscopy, as opposed to colectomy surgery. It is tempting to speculate that an up-regulation of EETs is a physiological response to contain inflammation, and that this protective mechanism may be impacted by disease progression.

**Fig 7:**
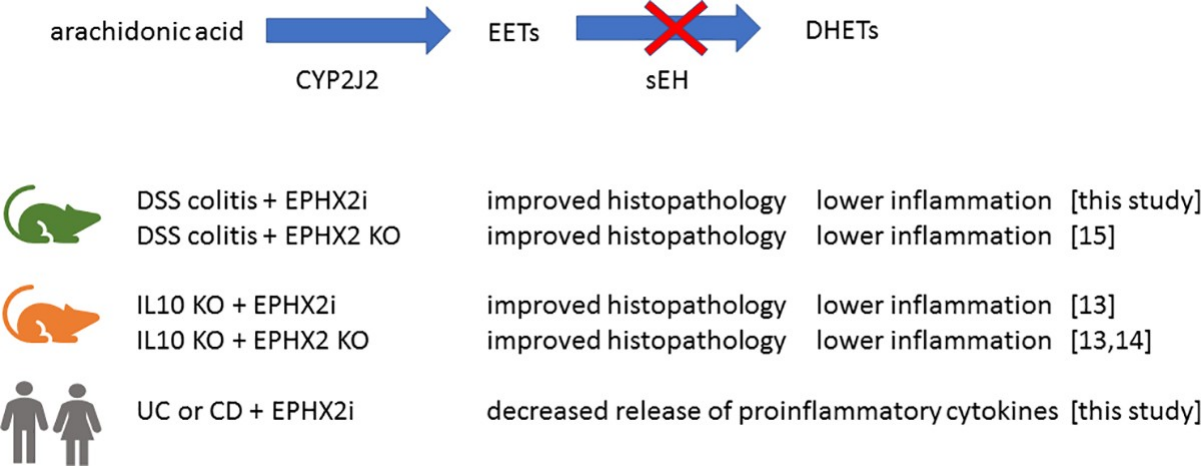
Summary of EPHX2i and knockout in mouse models and human disease explants. Similar protective effects on histology and inflammation were observed whether EPHX2 was decreased using chemical inhibitors or due to genetic deficiency. This was the case for both DSS-induced colitis and IL10 knockout mouse models. In colon explants from human patients, EPHX2i decreased production of inflammatory cytokines, compared to DMSO controls. This effect was seen in both UC and CD patient tissue.

In mice, either genetic deficiency of EPHX2 or treatment with a tool EPHX2i have been reported to attenuate chronic active IBD in IL10 (-/-) and in DSS-induced colitis models [13–15]. EPHX2 (-/-) /IL10(-/-) double knockout mice also displayed decreased precancerous dysplasia and tumor size, when compared to IL10(-/-) mice [15]. Both studies demonstrated histopathological protection of the colon and decreased production of inflammatory cytokines at the protein and mRNA levels were accompanied by EPHX2 decrease. A subsequent study examined the pathology of EPHX2 (-/-) mice compared to wild type, when subjected to DSS treatment [14]. Again, both histopathologic and IHC analyses showed that EPHX2-deficient mice were significantly protected, when compared to wild type. We designed the present study to strengthen the validation of our *in silico* observations and to fill mechanistic gaps in our understanding of EPHX2 biology, which might pave the way for clinical investigations in this space. Our study results demonstrate that EPHX2i treatment significantly improves DAI score, offers concurrent histopathologic protection of colon tissue and reduces the production of inflammatory markers in the DSS model. Fig 7 provides a summary of the evidence supporting EPHX2i treatment for IBD.

The fact that EPHX2 inhibition appears beneficial in both the DSS and IL10 knockout (KO) colitis models is particularly interesting, since they are driven by very different mechanisms [47]. IL10 KO mice develop colon inflammation characterized by infiltrate of lymphocytes, macrophages and neutrophils [48], initially driven by Th1 response [49]. DSS treatment disrupts the epithelial barrier, allowing luminal bacteria or their antigens to gain access to the mucosa. This inflammatory process develops even if T cells mediating adaptive immunity are absent [50,51]. It is also worth noting that intestinal protein and mRNA expression of CCL2 is higher in IBD patients compared to normal, which may contribute to chemoattraction of monocytes to the inflamed mucosa [52,53]. EPHX2 tool inhibitor t-AUCB blocks monocyte chemotaxis to CCL2 [54]. This mechanism also argues for protective effects of EPHX2 inhibition in IBD.

Whether an animal model fully translates to human disease is always a crucial question. One approach to assess translatability is to examine how subsequently approved small molecule or biological therapies have behaved in the pre-clinical model of choice. Since the DSS-colitis model is widely used, it was possible to find publications comparing several current therapies. Mesalazine, olsalazine and budesonide were weakly effective or ineffective, whereas 6-thioguanine (6-TG) and CsA were effective in the DSS model [35]. Infliximab, an anti-TNF antibody, was effective in the DSS model [55]. Altogether, our analysis of the available information on mechanisms that have shown efficacy in the DSS model bolstered our confidence in the selection of the model and appropriate comparator to examine the effect of our EPHX2 inhibitor *in vivo*.

In our study, the EPHX2 inhibitor performed at least as well as a low dose CsA treatment in the DSS model. CsA is an immunosuppressant often used to prevent rejection of organ transplants, and it has also been indicated as a treatment for psoriasis, rheumatoid arthritis and other immunological disorders. For the 30–40% of severe UC patients who are refractory to the first line treatment of intravenous corticosteroids, CsA treatment is occasionally indicated. However, serious adverse events due to opportunistic infections and nephrotoxicity have occurred [56], further highlighting the potential advantages of EPHX2 inhibition as a differentiated therapeutic approach.

A potential limitation of our current observations is the focus on the well characterized DSS model. While no perfectly translational models of IBD exist, animal models other than DSS could also have been considered [33]. For example, the DSS model is most closely related to UC, as is oxazolone-treatment, while the 2,4,6-trinitrobenzenesulfonic acid (TNBS) model is considered more representative of CD. Therefore, given the caveats that apply to the different animal models, we chose to further evaluate our hypothesis in human disease tissue, making our observations in the biopsies from both types of patients highly relevant and complementary to the observations made *in vivo* in the DSS model. Altogether, the data presented here, generated with a computational systems biology approach, an *in vivo* relevant animal model and in patient derived tissue, strongly suggest that inhibition of soluble epoxide hydrolase is a promising new mechanism to address the significant unmet need remaining for diseases such as UC and CD.

## Supporting information

S1 Fig Individual animal data for DSS-colitis experiment. To illustrate variation at the individual animal level, we present plots for DAI time-course (A), DAI AUC. (B), and Histopathology score (C). Jitter has been added to better distinguish overlapping data points. Groups were as follows: sham (no DSS) N = 6, DSS only N = 12, CsA treatment N = 6 and EPHX2i treatment N = 6. All groups except sham were dosed with DSS from Day 0 to Day 5. CsA and EPHX2i treatment groups were dosed from Day 0 to Day 9.
(TIF)

S2 Fig Colon Measurements at Experiment Conclusion. Mean values are plotted for each group. Error bars represent standard errors. (A) Colon length was decreased in DSS-treated mice, compared to the vehicle control. Both CsA and EPHX2i treatment partially restored colon length toward the value found in the vehicle control group. (B) The colon weight-to-length ratio is increased by DSS-treatment, but partially restored to normal by cyclosporine treatment. Unexpectedly, EPHX2i treatment did not reverse the weight-to-length ratio increase, but instead appeared to further increase the ratio.
(TIF)

S1 Table LCM-based disease signature from human patients obtained from GeneLogic. (XLSX)

S2 Table CMAP results for GSK2256294A. Shown are the statistically-interesting hits for the compound expression profile and IBD-related disease signatures. Column headers are as follows. Signature name was derived from the disease expression data source. Dose (in μM) was the compound concentration(s) tested (in triplicate). Mean Cmap score reflects the relative strength of association between compound profile and disease signature. Large negative scores imply a strong inverse correlation. Enrichment score is derived from the Kolmogorov-Smirnov statistic (10). P-value is derived from the distribution generated by 10,000 step permutation analysis. Specificity measures the uniqueness of the relationship between compound and disease signature (lower values are more unique). Signatures with better specificity is the rank order of the listed disease signature compared to the entire disease expression dataset. Cmap score distribution lists the actual scores for each of the triplicate repeats. Platform used for measuring expression was either L1000/Genometry or Illumina. Cell lines used were the following: Caco-2 human epithelial colorectal adenocarcinoma, FIBRO primary human fibroblasts, SAEC human small airway epithelial cells, KERAT primary human keratinocytes, HuSkM human skeletal muscle cells, MCF7 human breast cancer cell line. Disease (if known) from which the signature was derived. Signature biological tissue source. Species signature was derived from. GEO identifier for signature. PMID PubMed identifier if signature data has been published.
(XLSX)
