# Supplementary figures and images for: Preclinical evaluation of EPHX2 inhibition as a novel treatment for inflammatory bowel disease
Source: PLoS One. 2019 Apr 19;14(4):e0215033. doi: 10.1371/journal.pone.0215033 (PMC6474586; doi:10.1371/journal.pone.0215033)

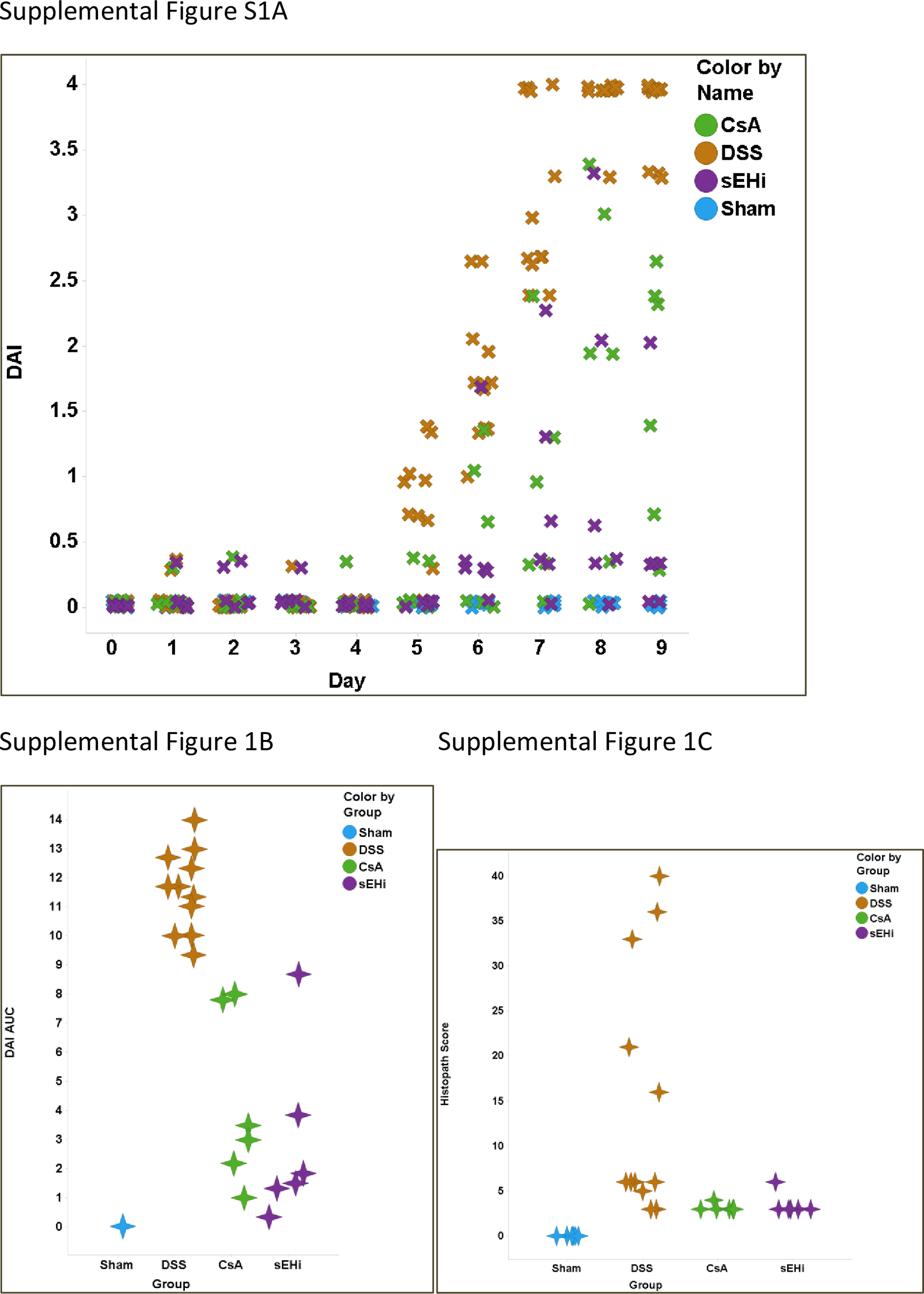

Supplement: S1 Fig — To illustrate variation at the individual animal level, we present plots for DAI time-course (A), DAI AUC. (B), and Histopathology score (C). Jitter has been added to better distinguish overlapping data points. Groups were as follows: sham (no DSS) N = 6, DSS only N = 12, CsA treatment N = 6 and EPHX2i treatment N = 6. All groups except sham were dosed with DSS from Day 0 to Day 5. CsA and EPHX2i treatment groups were dosed from Day 0 to Day 9. (TIF) [file pone.0215033.s001.tif]

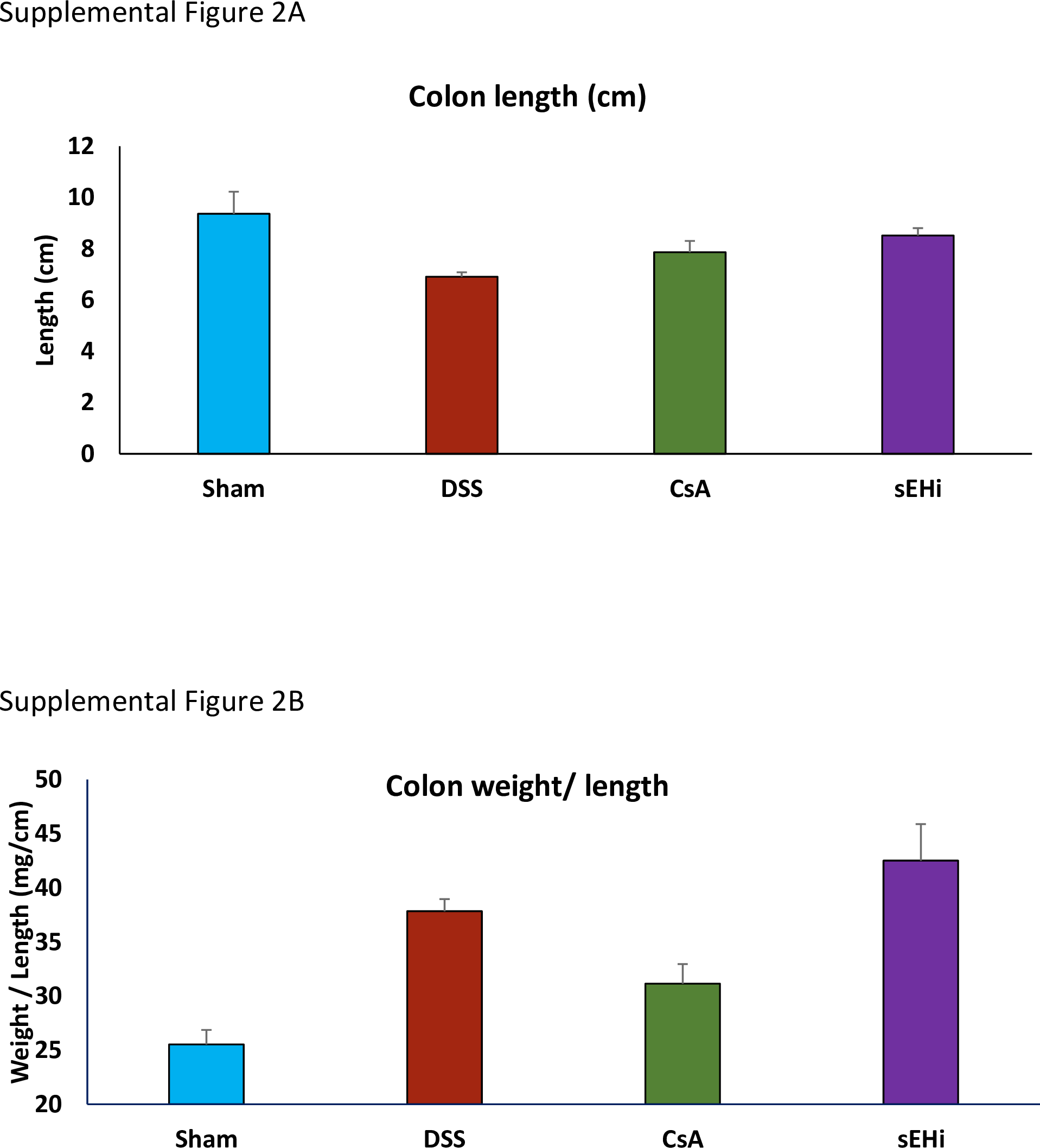

Supplement: S2 Fig — Mean values are plotted for each group. Error bars represent standard errors. (A) Colon length was decreased in DSS-treated mice, compared to the vehicle control. Both CsA and EPHX2i treatment partially restored colon length toward the value found in the vehicle control group. (B) The colon weight-to-length ratio is increased by DSS-treatment, but partially restored to normal by cyclosporine treatment. Unexpectedly, EPHX2i treatment did not reverse the weight-to-length ratio increase, but instead appeared to further increase the ratio. (TIF) [file pone.0215033.s002.tif]
